# Supplementary material for: In-field bioreactors demonstrate dynamic shifts in microbial communities in response to geochemical perturbations
Source: PLoS One. 2020 Sep 28;15(9):e0232437. doi: 10.1371/journal.pone.0232437 (PMC7521895; doi:10.1371/journal.pone.0232437)
Supplement: S1 File — (DOCX) [file pone.0232437.s001.docx]

**S1 Appendix**

Additional discussion of *Key taxa in changing communities*:

*Variovorax* is a wide-ranging soil microorganism that is often found in contaminated soils and includes species known to degrade toxic and/or complex organic contaminants^1^. It became very abundant in the reactors receiving contaminated water, reaching nearly 20% relative abundance on day 27 (Figure S9). In previous experiments with in-field bioreactors run at the Oak Ridge IFRC, *Aminobacter*, *Brevundimonas*, *Massilia*, and *Sphingobium* were significantly correlated to bioreactor nitrate concentrations ^2^. In this experiment, *Sphingobium* relative abundance significantly increased in control reactors and decreased in contaminated reactors post switch to GW706 water (Figure S9). This was unexpected, as *Sphingobium* are capable of assimilating nitrate and degrading chlorinated and aromatic hydrocarbons ^3^ and have been commonly detected in Oak Ridge IFRC 16S rRNA gene and 16S rRNA libraries in contaminated well ^4^. However, it likely that *Sphingobium* were unable to compete with *Ferribacterium* in contaminated reactors due to the other present geochemical forces. In previous in-field reactor experiments using FW305 water, *Sediminibacterium* alongside *Sphingobium* comprised significant portions (10-30%) of planktonic communities ^5^. In this experiment, *Sediminibacterium* comprised approximately 1-8% of relative abundance in all reactors, and peaked in abundance after t=18 correlating with the changing of sediment coupons (Fig. 10). *Parcubacteria* comprised 2-6% of relative abundance of the FW305 community as well as control reactors post switch (t=18) (Supplemental Figure S9). *Parcubacteria*, also known as the Candidate Phylum OD1, are often detected in groundwater and sediment communities at background sites at the Oak Ridge IFRC, but are often not abundant or not detected in contaminated wells [personal communication, Lauren Lui]. Members of the *Parcubacteria* have reduced genomes (<1 Mb) that are missing genes necessary for amino acid biosynthesis and DNA repair, indicating that they likely form symbiotic or parasitic relationships with other microorganisms for survival ^6^. Their low abundance in reactors receiving contaminated GW706 indicates either a direct geochemical forcing on their survival or indirect via other organisms they depend on. *Flectobacillus* represented a significant portion (10-45%) of planktonic microbial communities in all reactors pre-switch, and remained high (>10%) in all reactors post-switch (Figure S9). Members of *Flectobacillus* are filament-forming and capable of defending against grazing predators allowing them to flourish in low-nutrient conditions ^7^.

**References**

1 Satola, B., Wübbeler, J. H. & Steinbüchel, A. Metabolic characteristics of the species Variovorax paradoxus. *Applied microbiology and biotechnology* **97**, 541-560 (2013).

2 King, A. J. *et al.* Temporal Dynamics of In-Field Bioreactor Populations Reflect the Groundwater System and Respond Predictably to Perturbation. *Environ Sci Technol* **51**, 2879-2889, doi:10.1021/acs.est.6b04751 (2017).

3 Verma, H. *et al.* Comparative genomic analysis of nine Sphingobium strains: insights into their evolution and hexachlorocyclohexane (HCH) degradation pathways. *Bmc Genomics* **15**, doi:Artn 101410.1186/1471-2164-15-1014 (2014).

4 Spain, A. M. & Krumholz, L. R. Nitrate-Reducing Bacteria at the Nitrate and Radionuclide Contaminated Oak Ridge Integrated Field Research Challenge Site: A Review. *Geomicrobiology Journal* **28**, 418-429, doi:10.1080/01490451.2010.507642 (2011).

5 Christensen, G. A. *et al.* Use of in-field bioreactors demonstrate groundwater filtration influences planktonic bacterial community assembly, but not biofilm composition. *PLoS One* **13**, e0194663, doi:10.1371/journal.pone.0194663 (2018).

6 Nelson, W. C. & Stegen, J. C. The reduced genomes of Parcubacteria (OD1) contain signatures of a symbiotic lifestyle. *Front Microbiol* **6**, 713, doi:10.3389/fmicb.2015.00713 (2015).

7 Simek, K., Kasalicky, V., Hornak, K., Hahn, M. W. & Weinbauer, M. G. Assessing niche separation among coexisting Limnohabitans strains through interactions with a competitor, viruses, and a bacterivore. *Appl Environ Microbiol* **76**, 1406-1416, doi:10.1128/AEM.02517-09 (2010).

8 Christensen, G. A. *et al.* Determining factors that influence microbial community assembly using in-field bioreactors. (2017).


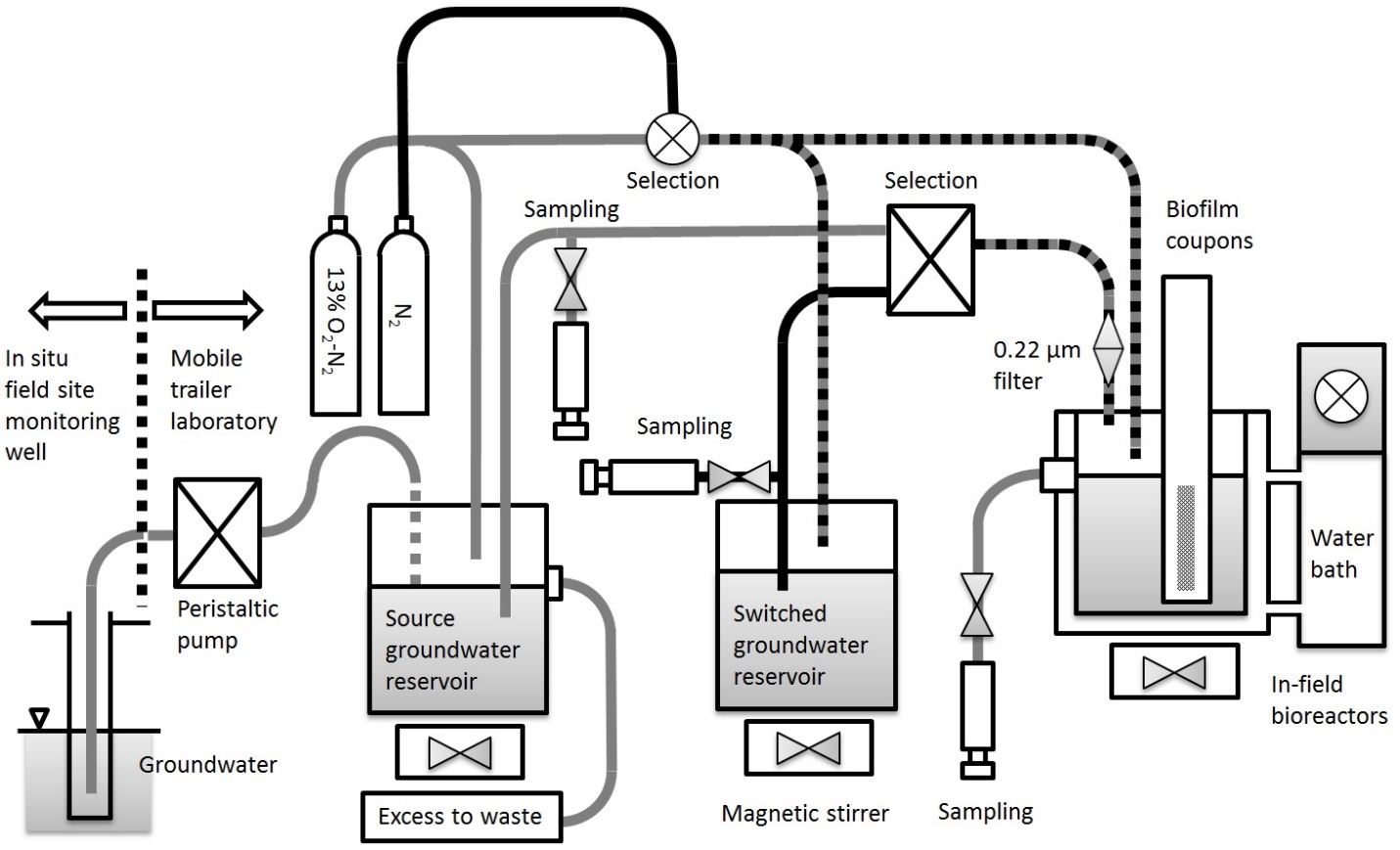


**
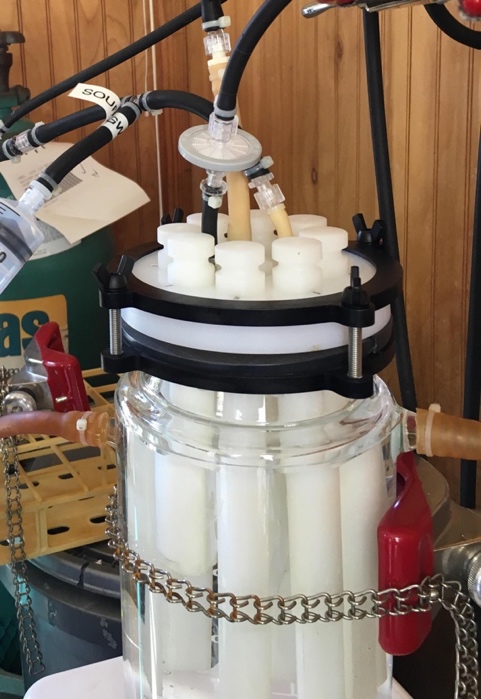
**

**S1 Fig**. A) Schematic diagram of experimental set-up of in-field bioreactors, and B) image of a 1L bioreactor unit, showing design of reactor and 8 removable sediment coupon inserts. Background groundwater was pumped directly from the well (FW305) into a source reservoir (200 mL min^-1^) prior to being filtered (0.22 µm) and pumped into triplicate bioreactors (0.22 mL min^-1^). Contaminated groundwater (GW706) was collected into sterile 2L glass carboys, filtered (0.22 µm), and pumped into triplicate bioreactors. Source reservoirs and bioreactors were temperature controlled using water baths (14 °C).

**S2 Fig.** Metal concentrations in filtered and unfiltered water collected from background (FW305 receiving) and contaminated (GW706 receiving) bioreactors, FW305 and GW706 groundwater well, and source (FW305) reservoir during duration of experiment. Error bars represent one standard error.

**S3 Fig**. Concentration of organic acids, A) formate and B) acetate, in reactors and source groundwater during duration of experiment.

**S4 Fig.** Total protein measured using BCA assay (top) and cell counts measured via acridine orange direct count (bottom) in unfiltered water collected from background (FW305 receiving) and contaminated (GW706 receiving) bioreactors, FW305 and GW706 groundwater well, and source (FW305) reservoir during duration of experiment. Error bars represent one standard error.


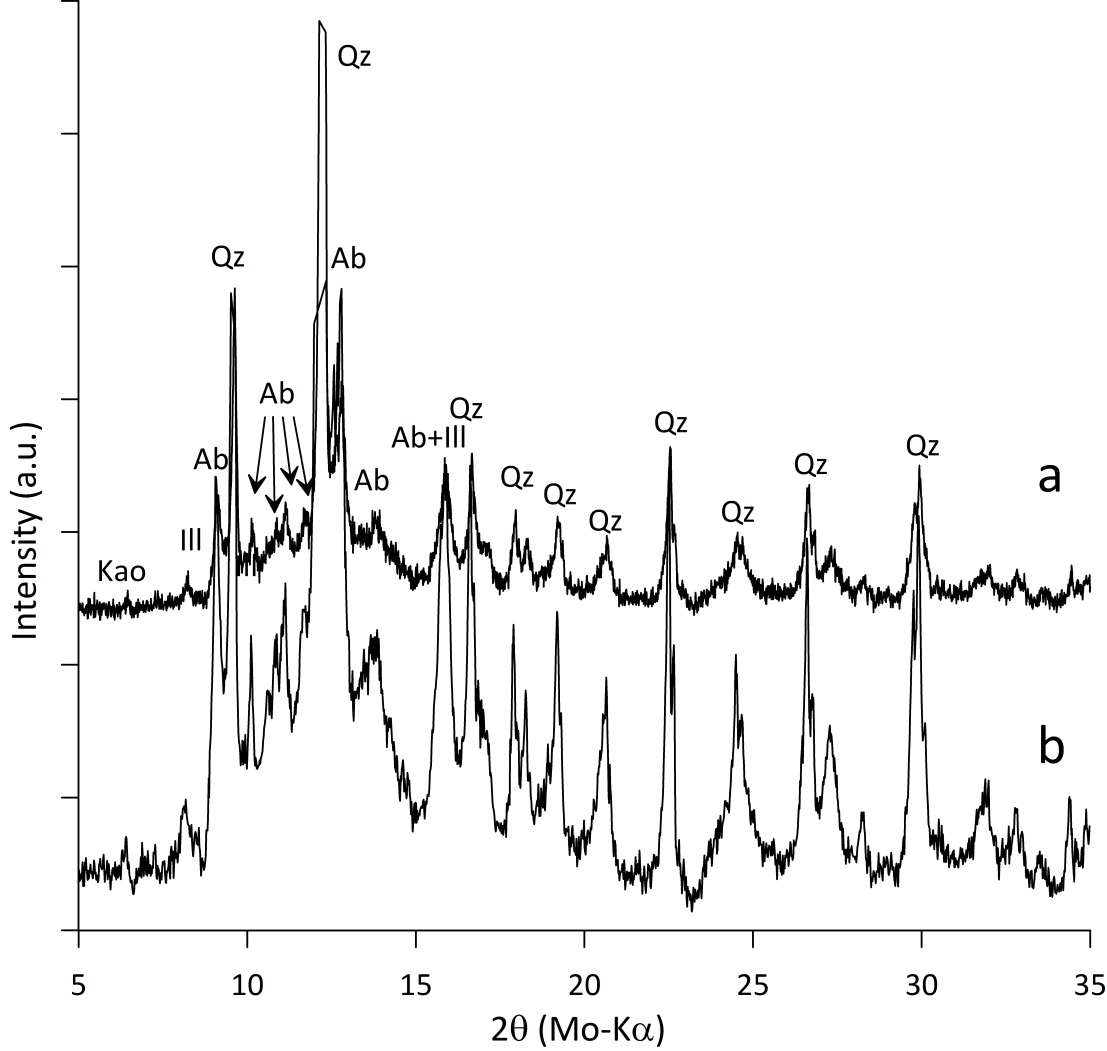


**S5 Fig.** The XRD patterns of a representative sample of core material used in the sediment coupon (b, FW305-06-06) showing the mineralogy compared to previous analysis of material from the same core (a, FW305-05-06, ^8^). The increased signal intensity suggests that the material used in this study was more mineralized with reduced amorphous or organic phases.


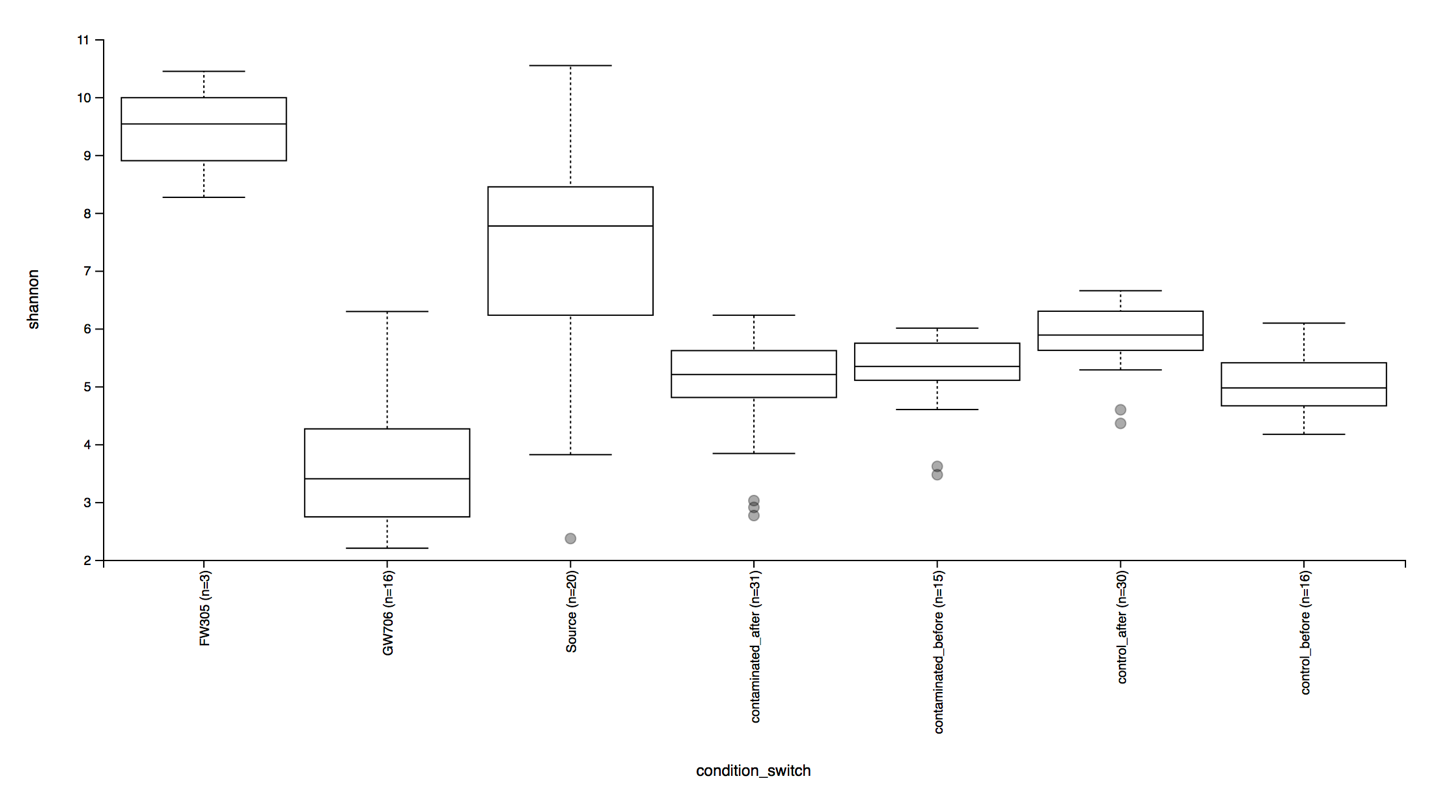


A

B


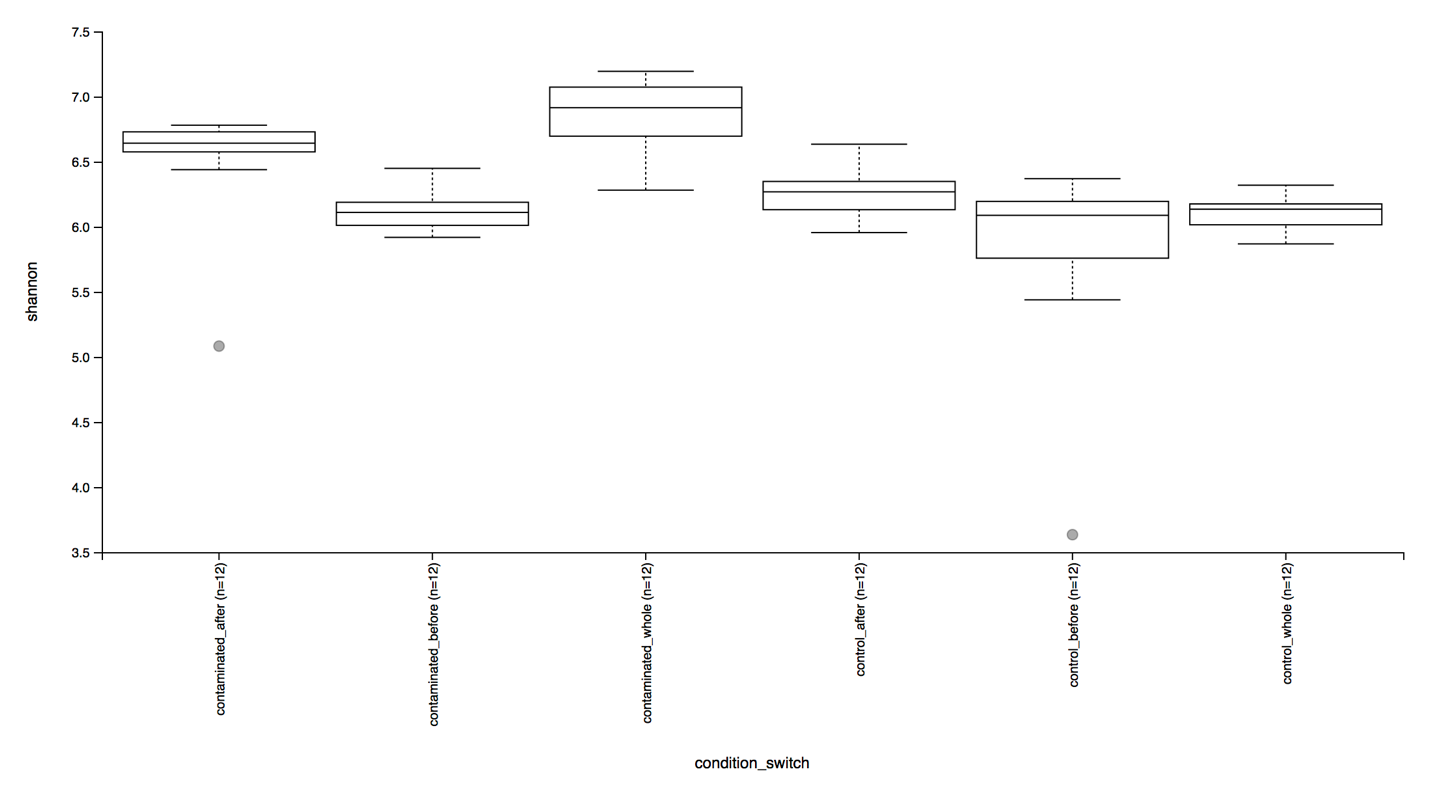


**S6 Fig.** Shannon diversity index of planktonic (a) and biofilm (b) samples grouped by condition. Planktonic microbial communities from wells and bioreactors receiving contaminated water were less diverse than control water samples, while no such clear trend was evident in biofilm samples.

**S7 Fig.** Significant (>0.5, p <0.05) differences in background groundwater well (FW305) and contaminated groundwater well (GW706) microbial communities collected on 0.2 µm filters.

**
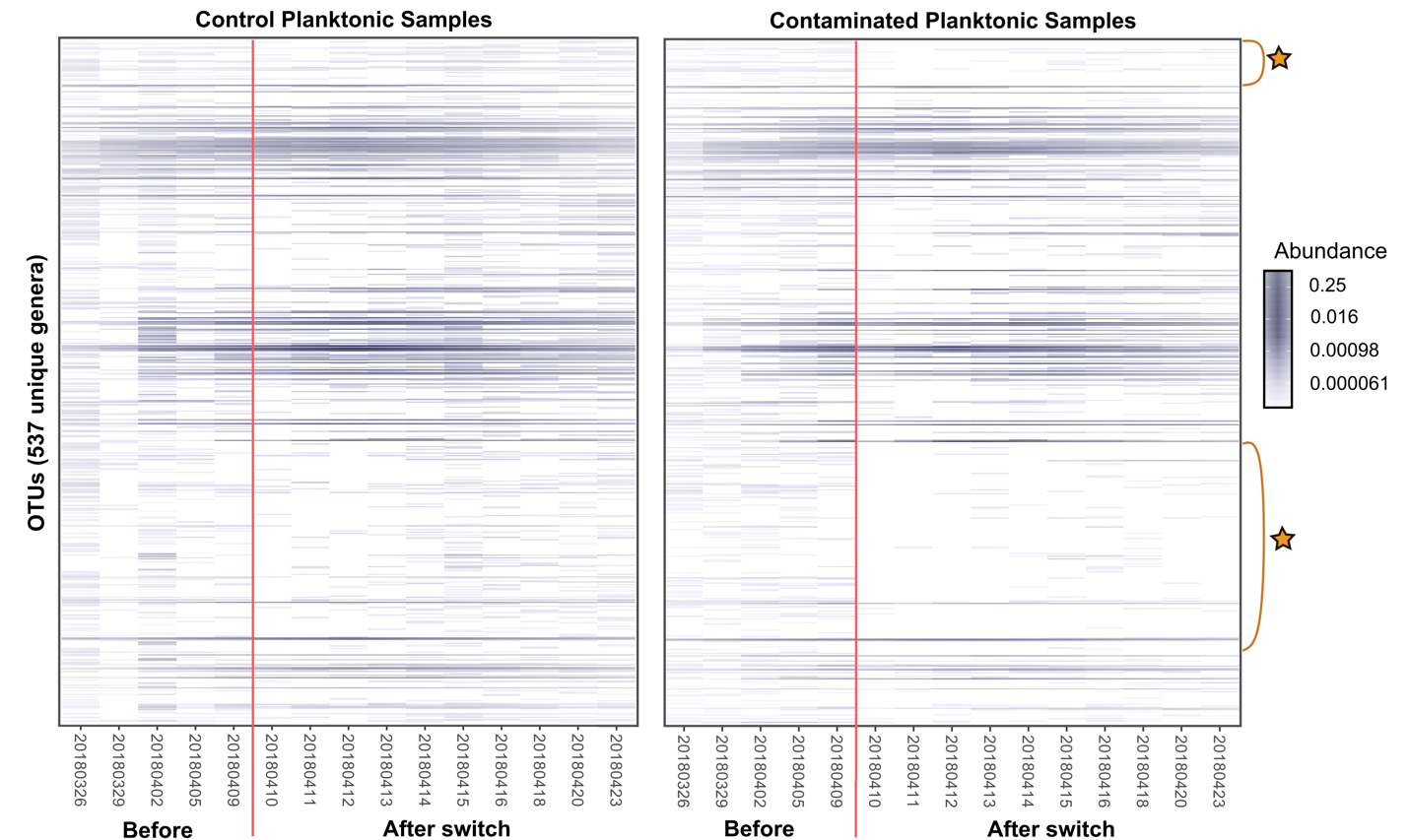
**

**S8 Fig.** Heatmap showing relative abundance of unique 16S rRNA amplicon sequence OTUs at the genus-level over time in planktonic communities from control (background) and contaminated reactors before and after switch from FW305 to GW706 water (t = 18) (in contaminated reactors only). Orange stars indicated genera where decrease in abundance was observed in contaminated reactors compared to control reactors.

**
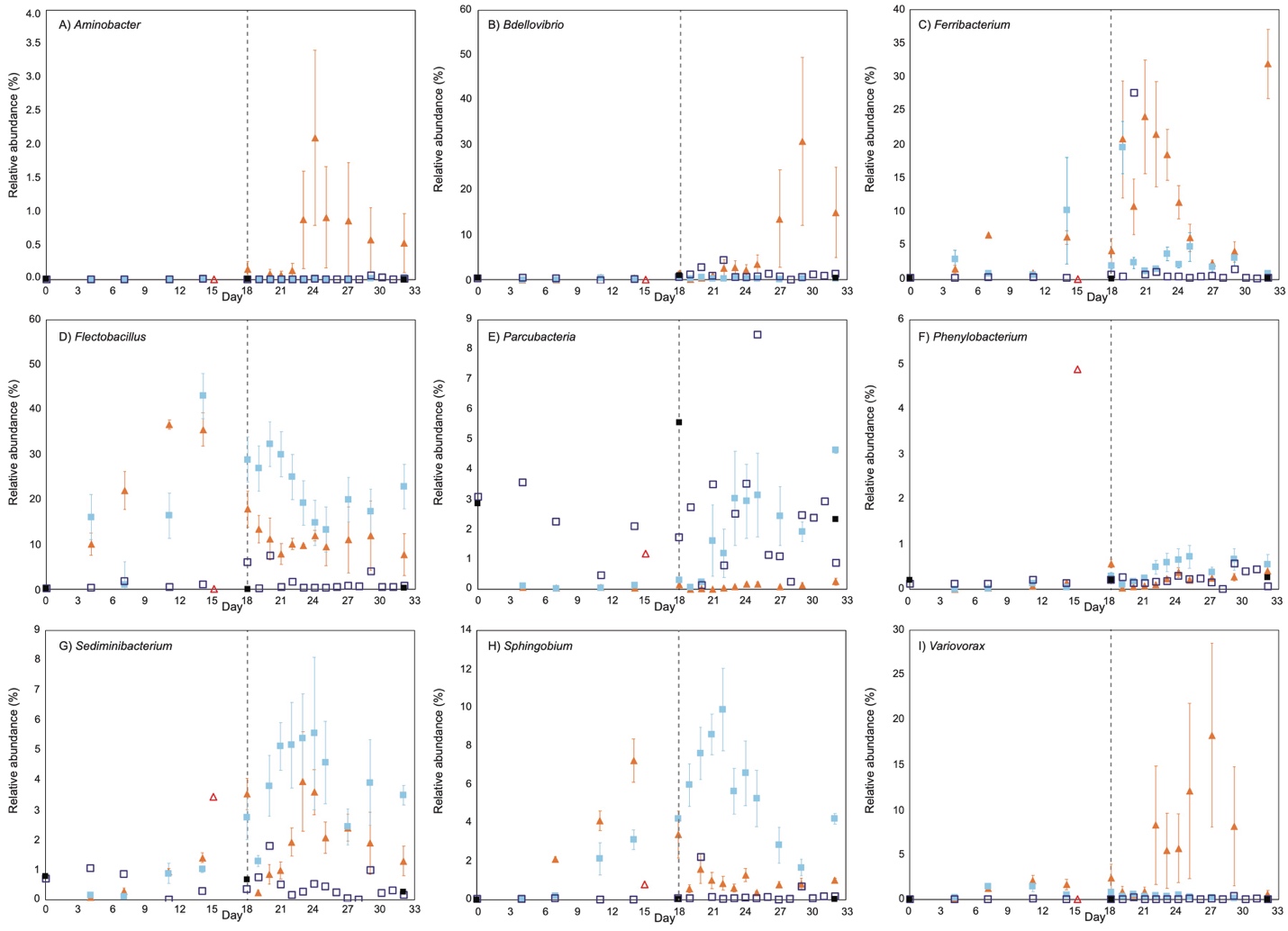
**

**S9 Fig.** Time series plots showing relative abundance of select genera in planktonic and well communities from background control (FW305-receiving) and contaminated (GW706-receiving after switch) bioreactors before and after switch (t = 18). Vertical dotted line indicates switch from control to contaminated water. Error bars represent one standard error of average from triplicate bioreactors.

**S10 Fig.** Nonmetric multidimensional scaling (NMDS) analysis of planktonic communities from each sampling point based on Bray-Curtis distance of log-transformed OTU abundance, overlaid with significant (p ≤ 0.001) factors (see Table S1). Number of dimensions = 2, number of permutations = 999, stress = 0.1225482. Environmental factors were overlaid using the “envfit” function in the R package vegan (Oksanen et al, 2018, https://CRAN.R-project.org/package=vegan).

**A**

**B**

**S11 Fig.** Random Forest models for planktonic (A) and biofilm (B) communities categorized by experimental condition. Overall model accuracy was 85.5% for planktonic samples and 94.4% for biofilm samples.

**S12 Fig**. PERMANOVA beta diversity analysis of Bray-Curtis distance between contaminated and control planktonic communities in bioreactors on day 18 (final day of growth prior to switching water sources) vs day 32 (final day of growth after switching water sources). Sample size = 12, number of groups = 4, permutations = 9999, pseudo-F = 2.77049, p-value = 0.0002.

**S13 Fig**. PERMANOVA beta diversity analysis of Bray-Curtis distance between contaminated and control sediment coupons incubated from day 0-18 (final day of growth prior to switching water sources) vs day 18-32 (final day of growth after switching water sources). Sample size = 48, number of groups = 4, permutations = 9999, pseudo-F = 8.25154, p-value = 0.0001.

**S1 Table.** Loadings of geochemical parameters to NMDS analysis of microbial communities.

* = p ≤ 0.05, ** = p ≤ 0.005, *** = p ≤ 0.001

|  | **NMDS1** | **NMDS2** | **r^2^** | **P-value** |  |
| --- | --- | --- | --- | --- | --- |
| **Acetate** | 0.81297 | 0.58231 | 0.0312 | 0.190 |  |
| **Aluminum** | 0.61373 | 0.78951 | 0.1929 | 0.001 | *** |
| **Ammonium** | -0.01639 | 0.99987 | 0.0501 | 0.062 | . |
| **Antimony** | 0.37883 | 0.92547 | 0.1893 | 0.001 | *** |
| **Arsenic** | 0.98850 | -0.15120 | 0.1389 | 0.001 | *** |
| **Bicarbonate** | -0.06153 | 0.99811 | 0.2745 | 0.001 | *** |
| **Cadmium** | 0.47706 | 0.87887 | 0.1196 | 0.001 | *** |
| **Calcium** | 0.00753 | 0.99997 | 0.2503 | 0.001 | *** |
| **Chloride** | 0.25144 | 0.96787 | 0.1996 | 0.001 | *** |
| **Chromium** | 0.34715 | -0.93781 | 0.0027 | 0.853 |  |
| **Cobalt** | 0.91004 | 0.41452 | 0.2428 | 0.001 | *** |
| **Conductivity** | 0.10681 | 0.99428 | 0.2226 | 0.001 | *** |
| **Copper** | 0.43984 | 0.89808 | 0.3953 | 0.001 | *** |
| **Day** | -0.75480 | -0.65596 | 0.0499 | 0.048 | * |
| **DO** | 0.85524 | -0.51824 | 0.4455 | 0.001 | *** |
| **Eh** | -0.74339 | -0.66886 | 0.0823 | 0.010 | ** |
| **Fluoride** | 0.40746 | 0.91323 | 0.3352 | 0.001 | *** |
| **Formate** | 0.35719 | 0.93403 | 0.0389 | 0.080 | . |
| **Iron** | -0.83511 | -0.55008 | 0.1091 | 0.002 | ** |
| **Lead** | 0.05048 | 0.99872 | 0.0797 | 0.010 | ** |
| **Lithium** | 0.96548 | -0.26048 | 0.0024 | 0.793 |  |
| **Magnesium** | 0.24473 | 0.96959 | 0.2265 | 0.001 | *** |
| **Manganese** | 0.98816 | 0.15345 | 0.3128 | 0.001 | *** |
| **Molybdenum** | 0.92212 | 0.38689 | 0.3743 | 0.001 | *** |
| **Nickel** | 0.16068 | 0.98701 | 0.0669 | 0.014 | * |
| **Nitrate** | 0.21949 | 0.97562 | 0.1978 | 0.001 | *** |
| **Nitrite** | 0.98248 | 0.18639 | 0.0204 | 0.221 |  |
| **OD600** | -0.87830 | -0.47810 | 0.0036 | 0.774 |  |
| **pH** | 0.96127 | 0.27560 | 0.7574 | 0.001 | *** |
| **Phosphate** | -0.26139 | -0.96523 | 0.1162 | 0.001 | *** |
| **Potassium** | 0.24477 | 0.96958 | 0.1818 | 0.001 | *** |
| **Protein** | 0.99419 | 0.10761 | 0.0891 | 0.008 | ** |
| **Sodium** | 0.23414 | 0.97220 | 0.2313 | 0.001 | *** |
| **Sulfate** | 0.24862 | 0.96860 | 0.1966 | 0.001 | *** |
| **Tin** | 0.64121 | 0.76737 | 0.1065 | 0.003 | ** |
| **Titanium** | 0.77841 | -0.62776 | 0.0184 | 0.305 |  |
| **Tungsten** | 0.98393 | 0.17854 | 0.0467 | 0.061 | . |
| **Uranium** | 0.17812 | 0.98401 | 0.2317 | 0.001 | *** |
| **Vanadium** | 0.99011 | -0.14026 | 0.2164 | 0.001 | *** |
| **Zinc** | -0.02111 | 0.99978 | 0.0283 | 0.179 |  |
